# Editorial: Genomic and metabolomic diversity in fruit plants: impacts of breeding techniques

**DOI:** 10.3389/fpls.2026.1822028

**Published:** 2026-04-14

**Authors:** Bruno Mezzetti, Björn Usadel, Sonia Osorio

**Affiliations:** 1 Department of Agricultural, Food and Environmental Sciences, Polytechnic University of Marche, Ancona, Italy; 2 Forschungszentrum Jülich, CEPLAS, BioSC, Institute of Bio- and Geosciences (IBG-4: bioinformatics), Jülich, Germany; 3 Institute for Biological Data Science, Heinrich-Heine-University Düsseldorf, Düsseldorf, Germany; 4 Instituto de Hortofruticultura Subtropical y Mediterránea “La Mayora”, Departamento de Biología Molecular y Bioquímica, Universidad de Málaga-Consejo Superior de Investigaciones Científicas (IHSM-UMA-CSIC), Málaga, Spain

**Keywords:** breeding, fruits, genetic diversity, genomics, metabolomics

The fruit and horticultural crop sector faces unprecedented challenges arising from climate instability, evolving consumer demands for nutritional quality, and the imperative to maintain genetic diversity whilst accelerating cultivar development. This Research Topic brings together contributions that explore how genomic and metabolomic approaches are reshaping our understanding of diversity in fruit-bearing plants and informing breeding strategies for crop improvement.

A central theme emerging from this Research Topic is the recognition that wild relatives and natural variation constitute invaluable reservoirs for crop enhancement. Pérez-Martín et al. provide a comprehensive review of how the woodland strawberry, *Fragaria vesca*, serves as a model system for dissecting the interplay between phenotypic plasticity and genetic adaptation. Their synthesis of metabolomic genome-wide association studies reveals how integrating metabolite profiling with genomic data can identify loci governing metabolic pathway diversity, offering a roadmap for exploiting wild germplasm to enhance crop resilience under changing environmental conditions.

The practical application of diversity screening is exemplified by two contributions addressing physiological and biochemical responses to genetic and environmental variation. Bharti et al. conducted an extensive phytochemical characterisation of 49 sweet pepper genotypes across developmental stages, documenting substantial variation in vitamin C, phenolic compounds, carotenoids, and anthocyanins. Their identification of strong correlations between antioxidant capacity and specific metabolites at different maturity stages provides actionable criteria for selecting genotypes with enhanced nutritional profiles. Similarly, Attar et al. evaluated drought tolerance mechanisms across 16 strawberry cultivars under controlled osmotic stress, revealing cultivar-specific responses in sugar accumulation, chlorophyll stability, and antioxidant activity. Their quantitative benchmarks for traits such as relative water content maintenance and photosynthetic efficiency under stress offer direct utility for breeding programmes targeting climate resilience.

Understanding the genetic architecture of reproductive and ornamental traits represents another dimension of diversity explored within this Topic. Wang et al. employed transcriptomic analysis combined with weighted gene co-expression network analysis to elucidate the molecular basis of reduced male fertility in loquat. Their demonstration that jasmonic acid biosynthesis and signalling pathways are suppressed in low-fertility genotypes, validated through metabolite quantification and hormone rescue experiments, provides mechanistic insight relevant to seedless fruit breeding. Complementing this molecular genetic approach, Hao investigated the inheritance patterns of corolla traits in Sinningia speciosa through extensive crossing programmes. The determination of heritability values and combining abilities for distinctive floral patterns establishes a quantitative framework for ornamental breeding that parallels approaches used in fruit crop improvement.

The translation of genomic knowledge into breeding outcomes remains a persistent challenge, critically examined by Pacheco-Ruiz et al. in their review of multi-omics integration in fruit agriculture. They articulate a sobering paradox: despite revolutionary advances in genomic technologies enabling allele identification within days and genome editing within weeks, the timeline for delivering improved cultivars to markets has not shortened over three decades. Their analysis identifies key bottlenecks including the integration of heterogeneous datasets, phenotyping limitations for complex traits, and tensions between technological innovation and biodiversity conservation. By framing fruit breeding as a data-to-decisions challenge, they outline systemic changes necessary for realising the potential of precision breeding approaches.

Collectively, these contributions illuminate both the opportunities and obstacles inherent in leveraging diversity for crop improvement. The studies span multiple species across temperate and subtropical climates, employ complementary methodological approaches from classical genetics to systems biology, and address traits ranging from stress tolerance to nutritional quality and reproductive biology. What unites them is recognition that meaningful progress requires integration across scales, from molecular mechanisms to whole-plant physiology, and across disciplines, from metabolomics to quantitative genetics.

Several priorities emerge for future research. First, the application of metabolomic GWAS approaches demonstrated in model systems must be extended to economically important crops where genomic resources are now increasingly available. Second, the metabolic markers identified for stress tolerance and nutritional quality require validation across environments and genetic backgrounds before deployment in marker-assisted selection. Third, the valley of death separating genomic discovery from cultivar release demands institutional and regulatory innovation alongside continued technical advancement.

This Research Topic demonstrates that the integration of genomic and metabolomic perspectives is no longer aspirational but operational in fruit plant research. The challenge ahead lies in translating this integrated understanding into breeding outcomes that serve producers, consumers, and the sustainability of agricultural systems.

